# Olmesartan-Induced Enteropathy: A Rare Case of Chronic Diarrhea

**DOI:** 10.1155/carm/2666671

**Published:** 2024-12-19

**Authors:** Sofia Emerenciano Gurgel, Kleyton Santos de Medeiros, Sarah Jane Lima de Paiva, José Gurgel Filho

**Affiliations:** ^1^Department of Neuroscience, University of Pittsburgh, Pittsburgh, Pennsylvania, USA; ^2^Instituto de Ensino, Pesquisa e Inovação, Liga Contra o Câncer, Natal, Rio Grande do Norte, Brazil; ^3^Department of Pathology, Getúlio Sales Diagnósticos, Natal, Rio Grande do Norte, Brazil; ^4^Department of Gastroenterology, Gastrocentro Natal, Natal, Rio Grande do Norte, Brazil

**Keywords:** case report, intestinal diseases, olmesartan

## Abstract

The case involves a 63-year-old hypertensive man, taking antihypertensive medication (olmesartan) for the previous two years, who sought medical attention due to voluminous diarrhea, with several episodes per day and weight loss of 10 kg. He was submitted to a series of diagnostic procedures without elucidation and empirical treatment with unsuccessful outcome. After hospitalization for clinical stabilization and for presenting with duodenal atrophy, obtained by duodenal biopsy associated with negative markers for celiac disease, the patient was diagnosed with suspected olmesartan-induced enteropathy, showing rapid improvement of diarrhea after the drug was withdrawn, with weight regain in 6 months and normalization of the duodenal histological picture after 10 months.

## 1. Introduction

The manuscript describes an interesting case of chronic diarrhea caused by olmesartan, a drug that was being used to treat high blood pressure. This clinical condition is rare and many gastroenterologists and most cardiologists that prescribe the medication are not aware of this side effect. As such, the report discusses the steps needed for diagnosis and is important to the medical community due to the persistent use of the drug and this side effect that can strongly impact a patient's life.

## 2. Case Presentation

A 63-year-old man with a history of hypertension and hypercholesterolemia presented to the gastroenterology clinic in March 2021 complaining of having nonbloody, nonmucoid, watery diarrhea more than 20 times a day, intermittent abdominal pain, nausea, and a weight loss of about 8 kg in 2 weeks. The patient had been using atorvastatina 20 mg and olmesartan 20 mg since 2019. He had not undergone any previous surgery. The patient sought medical help at the onset of the condition and was prescribed the antiparasitic agent nitazoxanide for 3 days and ciprofloxacin 500 mg bid for 7 days without any improvement in his condition. The patient's previous laboratory tests showed negative fecal occult blood and a negative stool culture. Clinical examination revealed no remarkable findings.

### 2.1. Investigations

Laboratory examination revealed a white cell count of 7420 × 10 9 cells/L, hemoglobin 14.4 g/dL, platelets 174 × 10^9^ cells/L cells/dL, sodium 138 meq/L, potassium 3.8 meq/L, chloride 104 meq/L, blood urea nitrogen 26 mg/dL, and creatinine 1.05 mg/dL. Liver enzymes were initially mildly elevated, alanine aminotransferase (ALT) 65 U/L (normal < 46), and aspartate aminotransferase (AST) 51 (normal < 35). Stool culture, *Clostridium difficile*, stool leukocytes, ova/parasites, and fecal calprotectin were negative but fecal occult blood initially tested positive.

**Figure 1:**
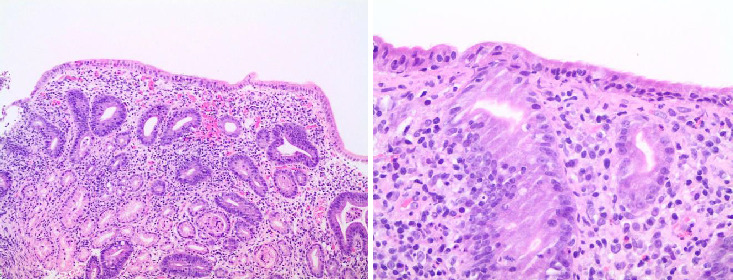
Histopathological image, June 2021.

Tissue transglutaminase IgA antibody, perinuclear antineutrophil cytoplasmic antibody, and antisaccharomyces cerevisiae antibodies were normal. Thyroid stimulating hormone (TSH) and C-reactive protein were normal. COVID-19 IgM and IgG were negative. Colonoscopy with serial biopsies was also normal (Figure 1). Ultrasonography revealed thickening of the gallbladder wall, echogenic bile, hydrops, signs of acalculous cholecystitis, and mild steatohepatitis. Gastroscopy showed a small hiatus hernia, features of gastritis including erythema, mucosal erosions (body and antrum), and the second part of the duodenum revealed mucosal atrophy with loss of folds. Biopsies of the second part of the duodenum showed intraepithelial lymphocytes and severe atrophic mucosa with complete loss of villi.

### 2.2. Treatment

The patient was prescribed tinidazole 2 g and instructed to start a gluten-free diet; however, the lack of clinical improvement caused him to seek the emergency medical unit, and he was hospitalized.

On the first day of hospitalization, the patient was given IV hydrocortisone, continued a gluten-free diet, and from then on, experienced considerable improvement in his clinical picture. On the day after admission, routine tests were performed, including serum cortisol, which showed 0.89 (10 < normal > 20 mcg/dL), persistent mild elevations of aminotransferases (ALT) 60 U/L (normal < 46), and aspartate aminotransferase (AST) 45 (normal < 35). During this first hospitalization, the patient maintained the antihypertensive drug olmesartan and atorvatatin. He was then discharged, given his improved condition after oral corticosteroids, but after this medication was withdrawn, severe diarrhea returned, and he was hospitalized once again.

### 2.3. Outcome and Follow-Up

During this second hospitalization, the medical team suspected that the condition could be olmesartan related because when the drug was discontinued, the symptoms gradually improved. After 3 months, the patient returned to the clinic and had gained 6 kg since the previous hospital admission. About 4 months after the initial disease, the patient underwent another endoscopy with biopsy of the second part of the duodenum, which revealed intraepithelial lymphocytes with moderate atrophic mucosa.

**Figure 2:**
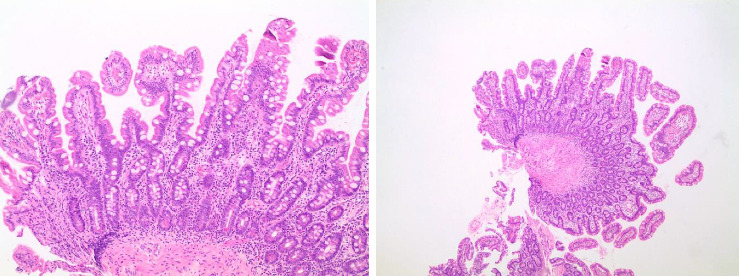
Histopathological image, April 2022.

After 11 months, he returned to the clinic symptom free with a weight gain of 9 kgs. A third gastroscopy was performed, and biopsy revealed normal mucosa without any signs of atrophic mucosa (Figure 2).

## 3. Discussion

Olmesartan is one of several angiotensin II receptor antagonists available for the treatment of hypertension. Since 2012, when Rubio-Tapia described 22 patients with OAE, 312 cases have been reported in the literature [1]. The main diagnostic finding was a histological picture of distal duodenum characterized by villous atrophy, massive intraepithelial lymphocyte infiltration, enhanced subepithelial collagen, and inflammation of the lamina propria in the absence of celiac disease. Although olmesartan-associated enteropathy (OAE) closely resembles celiac disease, it can be differentiated by negative tTG and endomysial antibodies. HLA-DQ2 and HLA-DQ8 heterodimers may be absent. In addition, there is no response to a gluten-free diet in olmesartan-associated enteropathy [2]. A gluten-free diet was tried without success, and HLA testing might have been more appropriate, but it was not performed in this case. Greater clinical vigilance regarding olmesartan-induced enteropathy could have contributed to switching to a gluten-free diet. The differential diagnosis is broad and includes autoimmune and drug-induced enteropathy, malignancies, infections, postinfectious enteropathy, and immunodeficient disorders [3]. In this case, the clinical data and the low serum cortisol result confused the medical team at the emergency room, leading to a misdiagnosis of an Addisonian crisis. This perception was maintained in view of the good clinical response to the intravenous corticosteroid. This condition was undone weeks after discharge with the gradual withdrawal of corticosteroids and new blood levels of serum cortisol that ruled out adrenal insufficiency, making it clear that the patient's response to corticosteroids is one of the possible characteristics of olmesartan-induced enteropathy.

It is very important to know the relationship between the drug and the development of chronic diarrhea. The mechanism for the induction of enteropathy is discussed in the literature. The long quiescent period between the onset of olmesartan and development of enteropathy is suggestive of cell-mediated immunity. Transforming growth factor-β (TGF-β) is a multifunctional cytokine that plays a role in gut hemostasis. Olmesartan has a higher affinity to block angiotensin II receptors (ATR) type-1, leaving angiotensin free to bind ATR type-2. This results in modulation of TGF-β, which in turn leads to histological changes in the small bowel mucosa [4].

In the present case, the first hospitalization day was marked by a quick response after the use of IV corticosteroids but low serum cortisol on the second day confused the medical team, and the possibility of a false positive was immediately evaluated. The patient improved and was referred to an endocrinologist, who ruled out adrenal dysfunction and discontinued corticosteroid therapy. The diarrhea returned and the dehydrated patient was hospitalized again. At this time, a review and discussion around the result of the duodenal biopsy raised the suspicion of OAE. The antihypertensive drug was discontinued and the patient gradually showed a significant improvement in his symptoms. After 4 months, the patient had normal bowel movements and a weight gain of 6 kg. A new endoscopy with biopsies of the duodenum showed intraepithelial lymphocytes with moderate atrophic mucosa, an improvement over the previous biopsy.

Liver injury has also been associated with OAE [5, 6]. Steroids may mitigate symptoms in 95% of the cases [7].

In our case, there was a small change in liver enzymes, which regressed after clinical resolution of the condition. Cases with gastric involvement have been reported [8]. In our case, the first gastroscopy revealed erosive gastritis in addition to duodenal atrophy. The microscopic findings revealed mild inflammation in the body and antrum of the stomach. The duodenum findings were extremely relevant. However, other microscopic findings are described in the literature, such as villous architectural distortion, increased intraepithelial lymphocytes, subepithelial collagen thickening, crypt apoptosis and crypt hyperplasia and chronic inflammation of the lamina propria with increased eosinophils [9]. There are also descriptions of colon involvement [3]. In the present case, colonoscopy and biopsy were normal. By contrast, in celiac disease, it can take years to achieve histological recovery, even under treatment [10]. Rapid mucosal recovery is described in OAE (median of 8 months after discontinuation of olmesartan) [11]. In this clinical case, a biopsy performed 11 months after drug withdrawal was normal, which was consistent with the literature. The question remains as to how many patients using the medication may have partial atrophy without showing clinical symptoms. We cannot draw conclusions, but everything indicates that OAE is very rare, as shown in a study with 2088 patients [12] or if histological changes are much more frequent than we imagine [13, 14]. Anyway, every patient with diarrhea using olmesartan should be aware of the possibility of developing OAE.

Although OAE closely resembles celiac disease, it can be differentiated by negative tTG, endomysial antibodies, and absence of HLA-DQ2 and HLA-DQ8 heterodimers.

The main diagnostic finding of the olmesartan-induced enteropathy are a histological picture of distal duodenum characterized by villous atrophy, massive intraepithelial lymphocyte infiltration, enhanced subepithelial collagen, and inflammation of the lamina propria in the absence of celiac disease.

## Data Availability

This is a case report in which the data are available in this material.

## Supporting Information

Supporting Document 1. Care Checklist.
